# Hyperuricemia Is a Risk Factor for the Onset of Impaired Fasting Glucose in Men with a High Plasma Glucose Level: A Community-Based Study

**DOI:** 10.1371/journal.pone.0107882

**Published:** 2014-09-19

**Authors:** Teruki Miyake, Teru Kumagi, Shinya Furukawa, Masashi Hirooka, Keitarou Kawasaki, Mitsuhito Koizumi, Yasuhiko Todo, Shin Yamamoto, Masanori Abe, Kohichiro Kitai, Bunzo Matsuura, Yoichi Hiasa

**Affiliations:** 1 Department of Gastroenterology and Metabology, Ehime University Graduate School of Medicine, Shitsukawa, Toon, Ehime, Japan; 2 Department of Community Medicine, Ehime University Graduate School of Medicine, Shitsukawa, Toon, Ehime, Japan; 3 Department of Public Health, Ehime University Graduate School of Medicine, Shitsukawa, Toon, Ehime, Japan; 4 Ehime General Health Care Association, Misake, Matsuyama, Ehime, Japan; Faculty of Biology, Spain

## Abstract

**Background:**

It is not clear whether elevated uric acid is a risk factor for the onset of impaired fasting glucose after stratifying by baseline fasting plasma glucose levels. We conducted a community-based retrospective longitudinal cohort study to clarify the relationship between uric acid levels and the onset of impaired fasting glucose, according to baseline fasting plasma glucose levels.

**Methods:**

We enrolled 6,403 persons (3,194 men and 3,209 women), each of whom was 18–80 years old and had >2 annual check-ups during 2003–2010. After excluding persons who had fasting plasma glucose levels ≥6.11 mM and/or were currently taking anti-diabetic agents, the remaining 5,924 subjects were classified into quartiles according to baseline fasting plasma glucose levels. The onset of impaired fasting glucose was defined as fasting plasma glucose ≥6.11 mM during the observation period.

**Results:**

In the quartile groups, 0.9%, 2.1%, 3.4%, and 20.2% of the men developed impaired fasting glucose, respectively, and 0.1%, 0.3%, 0.5%, and 5.6% of the women developed impaired fasting glucose, respectively (*P* trend <0.001). After adjusting for age, body mass index, systolic blood pressure, triacylglycerols, high density lipoprotein-cholesterol, creatinine, fatty liver, family history of diabetes, alcohol consumption, and current smoking, uric acid levels were positively associated with onset of impaired fasting glucose in men with highest-quartile fasting plasma glucose levels (adjusted hazard ratio, 1.003; 95% confidence interval, 1.0001–1.005, *P* = 0.041).

**Conclusions:**

Among men with high fasting plasma glucose, hyperuricemia may be independently associated with an elevated risk of developing impaired fasting glucose.

## Introduction

The prevalence of diabetes has been increasing worldwide, and the total number of people with diabetes has been projected to rise from 94 million in 2003 to 333 million in 2025 [1]. Those who have untreated diabetes can develop multiple complications, such as diabetic nephropathy and cardiovascular disease, and have reduced healthy life expectancies. Therefore, it is important to identify persons who are at a high risk of diabetes onset, and to prevent these persons from developing abnormal glucose intolerance.

Uric acid is the final oxidation product of purine catabolism. Elevated uric acid is considered to be a precursor of gout [2], one of the most common metabolic diseases, and is also related to the development of multiple complications in other diseases [3], [4]. With respect to glucose metabolism, some cross-sectional and longitudinal studies have found no association between uric acid levels and the risk of type 2 diabetes [5], [6] but other studies have reported their association [7]–[11]. Most notably, a meta-analysis by Kodama et al. found an association between uric acid levels and the development of type 2 diabetes [11]. To date, however, the majority of studies have not differentiated between men and women (including the study be Kodama et al.) [5], [8], [11]. Indeed, only a few studies include sex-specific analyses [7], [9], [10]. Yet, uric acid is metabolized differently in men and women because of the estrogen effect, which promotes the excretion of uric acid [12], [13]. Indeed, uric acid levels are generally higher in men.

The World Health Organization, International Diabetes Federation [14], and Japan Diabetes Society distinguish between normal and impaired fasting glucose [15] because impaired fasting glucose is independently associated with the onset of type 2 diabetes mellitus [14]–[18]. In addition, several research groups have reported that impaired fasting glucose is a risk factor for coronary artery disease [17], [19]. It is not known whether uric acid level is a risk factor for the onset of impaired fasting glucose, regardless of the baseline fasting plasma glucose level, which is itself a known risk factor for the onset of prediabetes and type 2 diabetes [20], [21].

This large, community-based longitudinal cohort study was designed to allow an epidemiologic assessment of the potential relationship between uric acid levels and the onset of impaired fasting glucose, as stratified by fasting plasma glucose levels at baseline. Ideally, the results of the present study will help clinicians to recognize the patients who are at greatest risk by identifying additional factors that could help to stratify patient risk.

## Patients and Methods

In accordance with the 1975 Declaration of Helsinki, *a priori* approval for the study was obtained from the Ehime University Hospital Research Ethics Board (Approval ID #110405, University hospital Medical Information Network ID: UMIN000011953), and all study procedures were conducted in accordance with guidelines on good clinical practices, as well as local ethical and legal requirements.

This retrospective, community-based, longitudinal cohort study began with a review of the medical records of 6,403 Japanese subjects (3,194 men and 3,209 women), whose ages ranged from 18 to 80 years, and who had undergone annual health check-ups at the Ehime General Health Care Association more than twice between April 2003 and March 2011. The annual health check-up included a record of the patient’s history of medical conditions and the medications prescribed for these conditions, as well as a physical examination and the measurement of anthropometric and routine biochemical variables. Body weight and height were measured while the subjects were clothed in light gowns without shoes, and the resulting measurements were used to calculate body mass index (BMI). Blood pressure measurements were performed with an automated sphygmomanometer while the subjects were seated. Blood samples were collected in the morning, after the subjects had been fasting for ≥10 h. These samples were used to (a) measure serum uric acid levels; (b) determine the patient’s risk of diabetes by measuring fasting plasma glucose levels; (c) assess the patient’s lipid profile, including triacylglycerols and high-density lipoprotein cholesterol levels; and (d) analyze renal function through creatinine levels. To examine the backgrounds of the study subjects, public health nurses asked all subjects to complete a questionnaire that assessed health-related behaviors prior to the patients’ health check-ups. This questionnaire included the frequencies and quantities of alcohol and cigarette consumption, and second-degree family history of diabetes. Alcohol consumers were defined as persons consuming alcohol at ≥20 g/day [22], [23]. Fatty liver was diagnosed based on a review of abdominal ultrasonography (Hitachi EUB-2000 or Hitachi Avius, Tokyo, Japan) by experienced technicians who were blinded to the subjects’ individual data. Two gastroenterologists (K. K. and K. K.) reviewed copies of all ultrasonography images to diagnose fatty liver disease. Of the 4 known criteria for fatty liver that can be identified from ultrasonography (hepatorenal echo contrast, liver brightness, deep attenuation, and vascular blurring) [24], evidence of 2 specific criteria (hepatorenal contrast and liver brightness) were required for diagnosis in this study.

**Figure 1:**
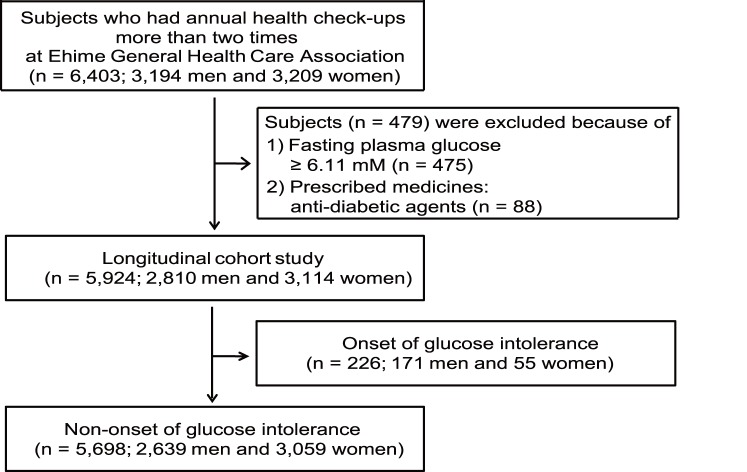
Recruitment flowchart for study participants.

After laboratory data and medical histories were assessed at the first check-up, 479 subjects were excluded from this study because they met at least one of the following exclusion criteria: (a) currently on a regimen of anti-diabetic agents (n = 88) and/or (b) fasting plasma glucose ≥6.11 mM (n = 475) (**Figure 1**). After excluding these subjects, we began our analysis of the remaining 5,924 subjects (2,810 men and 3,114 women) at Ehime University Hospital. The observation period lasted 3.45±1.88 years (3.38±1.81 years for men and 3.52±1.94 years for women) with a median of 3.01 years (range, 0.44–7.79 years). The median interval between the visits was 1.03 years (range, 0.44–7.23 years), with only 0.06% being less than 6 months and 14.2% being more than 2 years.

The eligible subjects were classified into quartiles (quartile 1, quartile 2, quartile 3, and quartile 4) according to their baseline fasting plasma glucose levels (Men: <5 mM, 5–5.22 mM, 5.23–5.49 mM, and 5.55–6.10 mM, respectively; Women: <4.66 mM, 4.66–4.87 mM, 4.88–5.15 mM, and 5.16–6.10 mM, respectively) and uric acid levels (Men: <327.1 µM, 327.1–368.6 µM, 368.7–416.4 µM, and >416.4 µM, respectively; Women: <220 µM, 220–249.9 µM, 250–279.6 µM, and >279.6 µM, respectively). The onset of impaired fasting glucose during the observation period was defined as a fasting plasma glucose level ≥6.11 mM at any health check-up.

All subjects were assigned a numerical code that was used throughout the study, and all data were stored in a secure database to maintain anonymity. All statistical analyses were performed using JMP version 11 software (SAS Institute Japan, Tokyo, Japan). One-way analysis of variance was used to analyze between-group differences in baseline characteristics, such as age, results of the physical examinations, and anthropometric and routine biochemical variables. The groups were defined according to fasting plasma glucose levels and uric acid levels. The chi-square test was used to analyze the presence or absence of fatty liver and health-related behaviors (such as alcohol consumption, cigarette consumption, and family history of diabetes). The Cochran–Armitage test was used to assess trends in the incidence rates of impaired fasting glucose onset according to fasting plasma glucose levels and uric acid levels. To explore the independent importance of each variable associated with the incident rate of impaired fasting glucose onset, we performed univariate and multivariate Cox proportional hazards regression analyses using forward likelihood ratio tests. The assumption of proportional hazards was assessed by including time-dependent covariates in the models; no indication of a violation was found. The following variables are known to affect glucose intolerance [25]–[29], and were included in the multivariate Cox regression models (from which we obtained adjusted hazard ratios of uric acid for the incidence of impaired fasting glucose onset): age, BMI, systolic blood pressure, triacylglycerols, high-density lipoprotein cholesterol, creatinine, fatty liver, family history of diabetes, alcohol drinking status, and current smoking status (Model 1). The remaining models (Models 2 and 3) included factors that were significant in the univariate analyses (*p*<0.05). Using a receiver–operating characteristic (ROC) curve analysis, the uric acid cutoff for predicting impaired fasting glucose onset was selected to maximize the calculated value of sensitivity - (1-specificity). All data are expressed as mean ± standard deviation, all *P*-values were 2-tailed, and *P*-values <0.05 were considered statistically significant.

## Results

### Baseline characteristics and the onset of impaired fasting glucose in each of the fasting plasma glucose and uric acid groups

The baseline characteristics of the study subjects are presented in **Tables 1** **–** **4**. For each of the subjects, complete data were available for all of the examined variables, with the exception of the BMI of 1 subject (0.017%). Among men, higher quartiles of fasting plasma glucose were associated with older subjects, as well as greater positive rates for metabolic and serum markers that are known to indicate metabolic disease (high BMI, systolic blood pressure, triacylglycerols, uric acid, and low high-density lipoprotein cholesterol), greater prevalence of fatty liver, and greater proportion of alcohol consumers (*P*<0.01 for each variable; **Table 1**). Among women, higher fasting plasma glucose groups had greater proportions of current smokers, as well as being associated with the same factors noted among men (*P*<0.01 for all variables; **Table 2**). The incidence rates of impaired fasting glucose in quartiles 1, 2, 3, and 4 were 0.9%, 2.1%, 3.4%, and 20.2% in men, respectively (**Table 1**), and 0.1%, 0.3%, 0.5%, and 5.6% in women, respectively (**Table 2**). In both sex-specific groups, the incidence rate of impaired fasting glucose increased significantly as the fasting plasma glucose level increased (*P-value for trend* <0.001).

**Table 1:** Men: baseline characteristics and the onset of impaired fasting glucose* according to fasting plasma glucose levels.

		Total (n = 2810)	Quartile 1 (n = 822)	Quartile 2 (n = 623)	Quartile 3 (n = 740)	Quartile 4 (n = 625)	*P*-value
Age	(years)	42.9±9.1	40±8.8	42.3±9.2	43.6±8.7	46.3±8.8	<0.001
Body mass index	(kg/m^2^)	23.7±3.1	23±2.9	23.6±3.1	23.9±3	24.3±3.3	<0.001
Systolic blood pressure	(mmHg)	117.3±14.7	114.6±14.6	115.4±14.3	119.2±14.3	120.4±14.7	<0.001
Creatinine	(µM)	77.5±11.2	77.2±10.1	77.8±11.6	77.7±12.5	77.1±10.6	0.596
Triacylglycerols	(mM)	1.53±1.17	1.37±0.04	1.51±0.05	1.59±0.04	1.71±0.05	<0.001
High-density lipoproteincholesterol	(mM)	1.56±0.39	1.61±0.42	1.54±0.4	1.54±0.36	1.52±0.39	<0.001
Uric acid	(µM)	369.3±71.3	363.3±71	367.4±68.6	374.3±73.3	373.3±71.3	0.007
Fatty liver		897/2810 (31.9%)	209/822 (25.4%)	198/623 (31.8%)	250/740 (33.8%)	240/625 (38.4%)	<0.001
Family history of diabetes		447/2810 (15.9%)	117/822 (14.2%)	95/623 (15.3%)	125/740 (16.9%)	110/625 (17.6%)	0.284
Current smoker		1175/2810 (41.8%)	13/33 (39.4%)	368/657 (56%)	886/1495 (59.3%)	368/625 (58.9%)	0.685
Alcohol drinker		1310/2810 (46.6%)	331/822 (40.3%)	268/623 (43%)	384/740 (51.9%)	327/625 (52.3%)	<0.001
Onset of impaired fasting glucose*		171/2810 (6.1%)	7/822 (0.9%)	13/623 (2.1%)	25/740 (3.4%)	126/625 (20.2%)	*P*-value for trend <0.001

Values are expressed as mean ± standard deviation.

Quartile 1: fasting plasma glucose <5 mM; quartile 2: fasting plasma glucose 5–5.22 mM; quartile 3: fasting plasma glucose 5.23–5.49 mM; quartile 4: fasting plasma glucose 5.55–6.10 mM.

For continuous values, differences among groups were assessed using one-way analysis of variance. The chi-square test was employed for comparisons of prevalence. The Cochran–Armitage test was used to assess the trend in the incidence rates of impaired fasting glucose onset according to the fasting plasma glucose groups.

*Onset of impaired fasting glucose was defined as a fasting plasma glucose level ≥6.11 mM.

**Table 2:** Women: baseline characteristics and the onset of impaired fasting glucose* according to fasting plasma glucose levels.

		Total (n = 3114)	Quartile 1 (n = 748)	Quartile 2 (n = 668)	Quartile 3 (n = 837)	Quartile 4 (n = 861)	*P*-value
Age	(years)	41.3±9.1	38.5±8.3	40.1±8.7	41.6±9.1	44.5±9	<0.001
Body mass index	(kg/m^2^)	21.4±3.1	20.4±2.4	20.8±2.7	21.5±3	22.7±3.7	<0.001
Systolic blood pressure	(mmHg)	108.4±15.4	103±13.1	105.9±13.3	108.9±14.5	114.7±17.1	<0.001
Creatinine	(µM)	56.4±8.4	56.4±8.1	56.3±8.1	56.4±8.4	56.3±8.8	0.963
Triacylglycerols	(mM)	0.88±0.52	0.76±0.37	0.82±0.42	0.88±0.45	1.03±0.68	<0.001
High-densitylipoprotein cholesterol	(mM)	1.96±0.43	2.02±0.42	1.98±0.4	1.94±0.44	1.9±0.43	<0.001
Uric acid	(µM)	254.9±53.6	247.5±49.7	249.7±50	254±51.4	266.4±59.7	<0.001
Fatty liver		347/3114 (11.4%)	32/748 (4.3%)	37/668 (5.5%)	100/837 (12%)	178/861 (20.7%)	<0.001
Family historyof diabetes		670/3114 (21.5%)	153/748 (20.4%)	141/668 (21.1%)	179/837 (21.4%)	197/861 (22.9%)	0.677
Current smoker		173/3114 (5.6%)	53/748 (7.1%)	42/668 (6.3%)	43/837 (5.1%)	35/861 (4.1%)	0.047
Alcohol drinker		338/3114 (10.9%)	58/748 (7.8%)	71/668 (10.6%)	90/837 (10.8%)	119/861 (13.8%)	0.002
Onset of impaired fastingglucose*		55/3114 (1.8)	1/748 (0.1)	2/668 (0.3)	4/837 (0.5)	48/861 (5.6)	*P*-value for trend<0.001

Values are expressed as mean ± standard deviation.

Quartile 1: fasting plasma glucose <4.66 mM; quartile 2: fasting plasma glucose 4.66–4.87 mM; quartile 3: fasting plasma glucose 4.88–5.15 mM; quartile 4: fasting plasma glucose 5.16–6.10 mM.

1 Body mass index value unavailable for 1 subject.

For continuous values, differences among groups were assessed using one-way analysis of variance. The chi-square test was employed for comparisons of prevalence. The Cochran–Armitage test was used to assess the trend in the incidence rates of impaired fasting glucose onset according to the fasting plasma glucose groups.

*Onset of impaired fasting glucose was defined as a fasting plasma glucose level ≥6.11 mM.

**Table 3:** Men: baseline characteristics and the onset of impaired fasting glucose* according to uric acid levels.

		Quartile 1(n = 699)	Quartile 2(n = 694)	Quartile 3(n = 777)	Quartile 4(n = 640)	*P*-value
Age	(years)	43.3±9.3	42.4±9	42.7±9.3	43.1±8.8	0.222
Body mass index	(kg/m^2^)	22.9±2.9	23.2±2.9	23.9±2.9	24.8±3.3	<0.001
Systolic blood pressure	(mmHg)	114.6±14.4	115.4±14	118±15.1	121.3±14.1	<0.001
Creatinine	(µM)	74.8±9.4	76.4±9.7	77.8±10.8	81±13.8	<0.001
Triacylglycerols	(mM)	1.23±0.79	1.43±0.85	1.55±1.04	1.97±1.71	<0.001
High-densitylipoprotein cholesterol	(mM)	1.63±0.39	1.56±0.4	1.55±0.4	1.47±0.37	<0.001
Fasting plasmaglucose	(mM)	5.2±0.38	5.24±0.38	5.24±0.36	5.25±0.38	0.047
Fatty liver		140/699(20%)	185/694(26.7%)	275/777(35.4%)	297/640(46.4%)	<0.001
Family historyof diabetes		106/699(15.2%)	104/694(15%)	115/777(14.8%)	122/640(19.1%)	0.102
Current smoker		316/699(45.2%)	309/694(44.5%)	301/777(38.7%)	249/640(38.9%)	0.014
Alcohol drinker		305/699(43.6%)	299/694(43.1%)	353/777(45.4%)	353/640(55.2%)	<0.001
Onset of impairedfasting glucose*		21/699(3%)	40/694(5.8%)	56/777(7.2%)	54/640(8.4%)	*P*-value fortrend <0.001

Values are expressed as mean ± standard deviation.

Quartile 1: uric acid <327.1 (µM); quartile 2: uric acid 327.1–368.6 µM; quartile 3: uric acid 368.7–416.4 µM; quartile 4: uric acid >416.4 µM.

For continuous values, differences among groups were assessed using one-way analysis of variance. The chi-square test was employed for comparisons of prevalence. The Cochran–Armitage test was used to assess the trend in the incidence rates of impaired fasting glucose onset according to the uric acid groups.

*Onset of impaired fasting glucose was defined as a fasting plasma glucose level ≥6.11 mM.

**Table 4:** Women: baseline characteristics and the onset of impaired fasting glucose* according to uric acid levels.

		Quartile 1(n = 710)	Quartile 2(n = 841)	Quartile 3(n = 691)	Quartile 4(n = 872)	*P*-value
Age	(years)	39.6±7.6	40.2±8.6	41.8±9.3	43.4±10	<0.001
Body mass index	(kg/m^2^)	20.5±2.5	20.9±2.7	21.4±3	22.6±3.7	<0.001
Systolic blood pressure	(mmHg)	105.9±13.7	106.3±14.1	109.1±16.5	112±16.2	<0.001
Creatinine	(µM)	53.7±7.8	565.4±7.5	57.1±7.9	58.8±9.2	<0.001
Triacylglycerols	(mM)	0.75±0.33	0.83±0.61	0.87±0.44	1.03±0.55	<0.001
High-densitylipoprotein cholesterol	(mM)	2.01±0.4	1.98±0.41	1.98±0.45	1.87±0.44	<0.001
Fasting plasmaglucose	(mM)	4.87±0.38	4.87±0.37	4.93±0.39	5±0.44	<0.001
Fatty liver		38/710(5.4%)	62/841(7.4%)	65/691(9.4%)	182/872(20.9%)	<0.001
Family historyof diabetes		160/710(22.5%)	182/841(21.6%)	135/691(19.5%)	193/872(22.1%)	0.523
Current smoker		36/710(5.1%)	40/841(4.8%)	40/691(5.8%)	57/872(6.5%)	0.39
Alcohol drinker		55/710(7.8%)	66/841(7.9%)	84/691(12.2%)	133/872(15.3%)	<0.001
Onset of impairedfasting glucose*		9/710(1.27%)	9/841(1.07%)	8/691(1.16%)	29/872(3.33%)	*P*-value fortrend 0.001

Values are expressed as mean ± standard deviation.

Quartile 1: uric acid <220 (µM); quartile 2: uric acid 220–249.9 µM; quartile 3: uric acid 250–279.6 µM; quartile 4: uric acid >279.6 µM.

1 Body mass index value unavailable for 1 subject.

For continuous values, differences among groups were assessed using one-way analysis of variance. The chi-square test was employed for comparisons of prevalence. The Cochran–Armitage test was used to assess the trend in the incidence rates of impaired fasting glucose onset according to the uric acid groups.

*Onset of impaired fasting glucose was defined as a fasting plasma glucose level ≥6.11 mM.

On the other hand, among men, higher quartiles of uric acid were associated with greater positive rates for metabolic and serum markers that are known to indicate metabolic disease (high BMI, systolic blood pressure, triacylglycerols, and fasting plasma glucose, and low high-density lipoprotein cholesterol), as well as elevated creatinine, greater prevalence of fatty liver, and greater proportions of current smokers and alcohol consumers (*P*<0.05 for each variable; **Table 3**). Among women, associations were observed for the same factors, excepting age and the proportion of current smokers (*P*<0.001 for all variables; **Table 4**). The incidence rates of impaired fasting glucose in quartiles 1, 2, 3, and 4 were 3%, 5.8%, 7.2%, and 8.4% in men, respectively (**Table 3**), and 1.27%, 1.07%, 1.16%, and 3.3%, in women, respectively (**Table 4**). In both sexes, the incidence rate of impaired fasting glucose increased significantly as the quartile of uric acid increased (*P-value for trend*<0.001).

### Risk factors for the onset of impaired fasting glucose

The results of the univariate analyses indicated that the following variables were significantly and positively associated with impaired fasting glucose onset in the men with the highest fasting plasma glucose at baseline (quartile 4): uric acid levels (hazard ratio [HR], 1.004; 95% confidence interval [CI], 1.002–1.007; *P*<0.001; **Table 5**) and previously reported risk factors for impaired fasting glucose (age, BMI, triacylglycerols, fasting plasma glucose, fatty liver, and current smokers). Risk factors for the onset of impaired fasting glucose in women were almost same as those observed in men, including uric acid: HR, 1.008; 95% CI, 1.003–1.013; *P*<0.001; **Table 6**). Notably, fatty liver had the high HR for each sex (Men: HR, 2.59; 95% CI, 1.818–3.724; *P*<0.001; Women: HR, 4.726; 95% CI, 2.678–8.413; *P*<0.001).

**Table 5:** Men: results of univariate analysis of risk factors for the onset of impaired fasting glucose* according to fasting plasma glucose levels.

		Quartile 1		Quartile 2		Quartile 3		Quartile 4	
		HR (95% CI)	*P* value	HR (95% CI)	*P* value	HR (95% CI)	*P* value	HR (95% CI)	*P* value
Age	(years)	1.02(0.933–1.108)	0.648	1.087(1.026–1.153)	0.005	1.023(0.977–1.071)	0.331	1.042(1.022–1.062)	<0.001
Body mass index	(kg/m^2^)	1.184(0.966–1.365)	0.096	0.962(0.784–1.151)	0.685	1.156(1.046–1.273)	0.005	1.063(1.012–1.11)	0.015
Systolic bloodpressure	(mmHg)	1.008(0.957–1.054)	0.755	1.049(1.013–1.084)	0.009	1.024(0.999–1.049)	0.057	1.005(0.992–1.017)	0.455
Creatinine	(µM)	0.983(0.91–1.061)	0.667	0.959(0.902–1.012)	0.144	0.95(0.909–0.992)	0.018	0.997(0.98–1.003)	0.69
Triacylglycerols	(mM)	1.42(0.652–2.304)	0.32	1.404(0.984–1.772)	0.059	1.212(0.982–1.377)	0.068	1.233(1.153–1.309)	<0.001
High-densitylipoprotein cholesterol	(mM)	0.729(0.379–1.372)	0.313	1.041(0.676–1.579)	0.855	0.672(0.484–0.927)	0.015	0.937(0.755–1.159)	0.552
Uric acid	(µM)	1.005(0.995–1.015)	0.303	1.006(0.998–1.014)	0.133	1.003(0.997–1.008)	0.316	1.004(1.002–1.007)	<0.001
Fatty liver	(%)	3.819(0.842–19.383)	0.081	0.618(0.138–2.031)	0.448	2.678(1.218–6.038)	0.015	2.59(1.818–3.724)	<0.001
Family historyof diabetes	(%)	0.945(0.05–5.536)	0.958	1.409(0.316–4.614)	0.615	1.186(0.395–2.929)	0.737	1.009(0.632–1.546)	0.969
Current smoker	(%)	0.999(0.197–4.533)	0.999	0.405(0.527–4.943)	0.405	1.303(0.586–2.873)	0.51	1.467(1.034–2.083)	0.032
Alcohol drinker	(%)	0.246(0.013–1.439)	0.131	1.114(0.358–3.361)	0.846	0.677(0.294–1.493)	0.336	1.179(0.83–1.683)	0.358

Quartile 1: fasting plasma glucose <5 mM; quartile 2: fasting plasma glucose 5–5.22 mM; quartile 3: fasting plasma glucose 5.23–5.49 mM; quartile 4: fasting plasma glucose 5.55–6.10 mM.

*Onset of impaired fasting glucose was defined as a fasting plasma glucose level ≥6.11 mM.

**Table 6:** Women: results of univariate analysis of risk factors for the onset of impaired fasting glucose* according to fasting plasma glucose levels.

		Quartile 1		Quartile 2		Quartile 3		Quartile 4	
		HR (95% CI)	*P* value	HR (95% CI)	*P* value	HR (95% CI)	*P* value	HR (95% CI)	*P* value
Age	(years)	1.176(0.91–1.647)	0.219	1.033(0.867–1.219)	0.695	1.051(0.939–1.173)	0.373	1.048(1.014–1.085)	0.006
BMI	(kg/m^2^)	0.909(0.438–1.672)	0.822	0.895(0.449–1.448)	0.715	1.096(0.777–1.457)	0.571	1.181(1.112–1.249)	<0.001
Systolic bloodpressure	(mmHg)	0.987(0.825–1.131)	0.862	1.043(0.942–1.138)	0.386	1.042(0.974–1.108)	0.222	1.011(0.996–1.026)	0.152
Creatinine	(µM)	1.081(0.836–1.304)	0.503	0.947(0.768–1.119)	0.559	0.907(0.784–1.035)	0.151	0.987(0.953–1.021)	0.46
Triacylglycerols	(mM)	0.745(0.00007–4.856)	0.914	0.661(0.003–7.591)	0.825	2.112(0.646–4.083)	0.165	1.339(1.12–1.503)	0.004
High-densitylipoprotein cholesterol	(mM)	2.359(0.02–149.116)	0.704	8.258(0.406–133.854)	0.158	0.713(0.056–5.618)	0.767	0.162(0.071–0.356)	<0.001
Uric acid	(µM)	0.978(0.954–1.011)	0.165	0.997(0.971–1.023)	0.81	1.015(0.998–1.031)	0.079	1.008(1.003–1.013)	<0.001
Fatty liver	(%)	Not calculated		Not calculated		Not calculated		4.726(2.678–8.413)	<0.001
Family historyof diabetes	(%)	Not calculated		4.07(0.161–102.825)	0.339	Not calculated		2.297(1.268–4.066)	0.007
Current smoker	(%)	Not calculated		Not calculated		5.294(0.261–41.538)	0.218	1.745(0.423–4.773)	0.389
Alcohol drinker	(%)	Not calculated		Not calculated		6.328(0.756–52.984)	0.084	1.435(0.623–2.905)	0.371

Quartile 1: fasting plasma glucose <4.66 mM; quartile 2: fasting plasma glucose 4.66–4.87 mM; quartile 3: fasting plasma glucose 4.88–5.15 mM; quartile 4: fasting plasma glucose 5.16–6.10 mM.

*Onset of impaired fasting glucose was defined as a fasting plasma glucose level ≥6.11 mM.

### Uric acid and risk factors for the onset of impaired fasting glucose

Among men, the adjusted hazard ratio (aHR) from Model 1 indicated a significant positive association between uric acid levels and the onset of impaired fasting glucose for the highest quartile of fasting plasma glucose (quartile 4: aHR, 1.003; 95% CI, 1.0001–1.005; *P* = 0.041; **Table 7**). Model 2 included adjustments for variables found to be significant in univariate analyses, revealing no significant association between uric acid levels and impaired fasting glucose onset in quartile 4 (aHR, 1.002; 95% CI, 0.99999–1.005; *P* = 0.051). Model 3 resembled Model 2 except that fatty liver was excluded. Model 3 indicated a significant positive association between uric acid levels and the onset of impaired fasting glucose in quartile 4 (aHR, 1.003; 95% CI, 1.0002–1.005; *P* = 0.034). Among women, none of the models revealed significant associations between uric acid levels and the onset of impaired fasting glucose in the highest quartile of baseline fasting glucose (**Table 8**).

**Table 7:** Men: associations between uric acid level (µM) and the onset of impaired fasting glucose* for high plasma glucose levels.

Fasting plasmaglucose at baseline	5.16–6.10 (mM) (Quartile 4)		
	aHR	(95% CI)	*P*-value
Model 1	1.003	(1.0001–1.005)	0.041
Model 2	1.002	(0.99999–1.005)	0.051
Model 3	1.003	(1.0002–1.005)	0.034

The aHR is expressed per µM increment in uric acid value.

Model 1 was adjusted for all variable that were previously reported to be associated with metabolic disease or risk factors for the onset of glucose intolerance: age (years), BMI (kg/m^2^), systolic blood pressure (mmHg), triacylglycerols (mM), high-density lipoprotein cholesterol (mM), creatinine (µM), fatty liver (%), family history of diabetes (%), alcohol drinking status (%), and current smoking status (%).

Model 2 was adjusted for all factors that were significant in univariate analyses: age, BMI, triacylglycerols, fatty liver, and current smoking status.

Model 3 was adjusted for all factors that were significant in univariate analyses, excluding fatty liver: age, BMI, triacylglycerols, and current smoking status.

*Onset of impaired fasting glucose was defined as a fasting plasma glucose level ≥6.11 mM.

aHR, adjusted hazard ratio; CI, confidence interval; BMI, body mass index.

**Table 8:** Women: associations between uric acid level (µM) and the onset of impaired fasting glucose* on high plasma glucose level.

Fasting plasmaglucose at baseline	5.16–6.10 (mM) (Quartile 4)		
	aHR	(95% CI)	*P*-value
Model 1	1.001	(0.996–1.006)	0.799
Model 2	1.001	(0996–1.005)	0.824

aHR expressed per µM increment in uric acid value.

Model 1 was adjusted for all variable that were previously reported to be associated with metabolic disease or risk factors for the onset of glucose intolerance: age (years), BMI (kg/m^2^), systolic blood pressure (mmHg), triacylglycerols (mM), high-density lipoprotein cholesterol (mM), creatinine (µM), fatty liver (%), family history of diabetes (%), alcohol drinking status (%), and current smoking status (%).

Model 2 was adjusted for all factors that were significant in univariate analyses: age, BMI, triacylglycerols, high-density lipoprotein cholesterol, fatty liver, and family history of diabetes.

*Onset of impaired fasting glucose was defined as a fasting plasma glucose level ≥6.11 mM.

aHR, adjusted hazard ratio; CI, confidence interval; BMI, body mass index.

### Evaluation of uric acid level as a predictive factor for the onset of impaired fasting glucose

Uric acid was identified as a significant risk factor for impaired fasting glucose onset among men with the highest quartile of baseline fasting plasma glucose. Further, we estimated the cutoff level that appeared optimal for predicting impaired fasting glucose onset. We evaluated the rate of subjects with a high uric acid level who developed impaired fasting glucose to determine the associated positive predictive value (PPV) for forecasting impaired fasting glucose onset. Similarly, we evaluated the rate of non- impaired fasting glucose subjects with a low impaired fasting glucose level was evaluated to determine the negative predictive value (NPV) of impaired fasting glucose for identifying subjects who would not develop impaired fasting glucose. The area under receiver–operating characteristic curve, cutoff level, sensitivity, specificity, PPV, NPV, and diagnostic accuracy for predicting impaired fasting glucose were 0.599 (95% CI: 0.559–0.637), 368.8 µM, 61.9%, 52.7%, 24.8%, 84.6%, and 54.6%, respectively.

## Discussion

We conducted this large, community-based longitudinal cohort study to examine the sex-specific associations between uric acid levels and the onset of impaired fasting glucose, according to baseline fasting plasma glucose levels in both sexes. Our findings indicate that hyperuricemia is a significant risk factor for the onset of impaired fasting glucose among men and women with high baseline fasting plasma glucose levels. The association remained significant for men after adjusting for potential confounders.

Recently, several studies have examined the associations between uric acid and the onsets of prediabetes and type 2 diabetes. To provide data for their meta-analysis, Kodama et al. searched Medline (31 March from 1966 to 2009) and Embase (31 March from 1980 to 2009) for observational cohort studies that examined the association between uric acid and the risk of type 2 diabetes onset [11]. Kodama et al. then used meta-regression analyses to investigate the effects of individual study characteristics on the association between uric acid level and type 2 diabetes risk. Based on their evaluation of 11 cohort studies (42,834 participants), Kodama et al. suggested that uric acid level is positively associated with the development of type 2 diabetes, regardless of the characteristics of individual studies. To assess whether elevated uric acid levels could predict impaired fasting glucose and type 2 diabetes, Yamada et al. examined 6,408 men and 5,309 women who had voluntarily undergone annual health checkups at the Okazaki City Medical Association Public Health Center in 2000 and in 2005 [7]. According to multiple logistic-regression analyses that adjusted for age, parental history of diabetes, BMI, hypertension, fatty liver, hypertriglyceridemia, alcohol consumption, and smoking status, Yamada et al. reported that elevated uric acid levels were only predictive of impaired fasting glucose and type 2 diabetes in Japanese women. However, the authors did not examine the effects of fasting plasma glucose at baseline. Moreover, they only examined 2 points in time (in 2000 and in 2005), even though participants may have undergone health checkups annually, and may have received interventions as a result.

Several potential mechanisms could explain the association between uric acid level and the onset of impaired fasting glucose. In experimental studies, uric acid levels were reported to induce insulin resistance by inhibiting the bioactivity of nitric oxide [30], which is essential for insulin-stimulated glucose uptake in skeletal muscle [31], and by promoting the secretion of inflammatory factors and adipocytokine [32]. Recently, several studies reported that xanthine oxidase (the enzyme involved in uric acid production) does not only produce reactive oxygen species, but also activates nuclear factor-kappa B [33], [34] and induces inflammation. These responses might cause insulin resistance. In addition, insulin resistance leads to hyperinsulinemia, which increases uric acid concentrations by reducing renal uric acid secretion [35] and accumulating substrates for uric acid production [36]. This possible mechanism, which links uric acid and insulin resistance, could explain why the combination of hyperuricemia and high baseline fasting plasma was a risk factor for the onset of impaired fasting glucose in the present study.

The primary strengths of our study were its investigation of the general population, and the completeness of the data for all variables, with the exception of only 1 missing data point. On the other hand, there are several limitations to our study. First, only 226 subjects (171 men and 55 women) showed impaired fasting glucose onset out of 5,924 participants (2,810 men and 3,114 women). This relatively low rate may explain the lack of a significant association between uric acid and impaired fasting glucose onset in women as well as uric acid and impaired fasting glucose onset in men when including fatty liver as a confounder. Uric acid may be a predictor in both sexes to different degrees, as could be revealed by a future study with a larger number of subjects. Second, the exclusion of a large part of the population in the upper range of fasting plasma glucose values (higher than 6.1 mM) may have limited the range of the relationship under study, possibly obscuring a wider relationship between fasting plasma glucose and uric acid. Third, we were only able to collect data annually; therefore, data collection was not truly continuous. Forth, our study relied on self-reported information for several of the investigated factors. Therefore, misreported data could have biased our findings. Fifth, we did not examine the consumption of animal protein, which is regarded as high-protein diet and associated with increased intake of purines [37]. The different habits of animal protein consumption between women and men may have affected our results. Sixth, we did not examine the glucocorticoids during the awaking process, which has a considerable influence on fasting glucose. The rise of glucocorticoids increases glycogen hydrolysis in the liver, increasing its glucose output and eliciting temporal hyperglycemia, which may be confused by diabetes. Finally, we investigated a solely Japanese population, which could limit the generalizability of our results to other populations. To correct these limitations, future studies should use a prospective validation design, and investigate diverse populations that present at various outpatient medical practices.

Despite these limitations, our study produced several noteworthy results. In particular, our findings suggest that hyperuricemia is a risk factor for the onset of impaired fasting glucose among persons with high baseline fasting plasma glucose. To help prevent the onset of impaired fasting glucose and the development of further complications, we should therefore pay careful attention to both uric acid levels and fasting plasma glucose levels, especially in subjects with hyperuricemia. It might be possible to prevent impaired fasting glucose and the development of further complications by treating both elevated fasting plasma glucose and hyperuricemia, although randomized studies would be necessary to validate this suggestion.

## References

[1] Yoon KH, Lee JH, Kim JW, Cho JH, Choi YH, et al (2006) Epidemic obesity and type 2 diabetes in Asia. Lance 368: 1681e8. 10.1016/S0140-6736(06)69703-1 17098087

[2] Roubenoff R, Klag MJ, Mead LA, Liang KY, Seidler AJ, et al (1991) Incidence and risk factors for gout in white men. J Am Med Assoc 266: 3004–3007. 1820473

[3] Baker JF, Krishnan E, Chen L, Schumacher HR (2005) Serum uric acid and cardiovascular disease: recent developments, and where do they leave us? Am J Med 118: 816–826. 16084170 10.1016/j.amjmed.2005.03.043

[4] Li Y, Xu C, Yu C, Xu L, Miao M (2009) Association of serum uric acid level with non-alcoholic fatty liver disease: a cross-sectional study. J Hepatol 50: 1029–1034. 19299029 10.1016/j.jhep.2008.11.021

[5] Taniguchi Y, Hayashi T, Tamura K, Endo G, Fujii S (2001) Serum uric acid and the risk for hypertension and Type 2 diabetes in Japanese men: Osaka Health Survey. J Hypertens 19: 1209–1215. 11446710 10.1097/00004872-200107000-00005

[6] Oda E, Kawai R, Sukumaran V, Watanabe K (2011) Uric acid is positively associated with metabolic syndrome but negatively associated with diabetes in Japanese men. Intern Med 48: 1785–1791. 10.2169/internalmedicine.48.2426 19834269

[7] Yamada T, Fukatsu M, Suzuki S, Wada T, Joh T (2011) Elevated serum uric acid predicts impaired fasting glucose and type 2 diabetes only among Japanese women undergoing health checkups. Diabetes Metab 37: 252–258. 21377910 10.1016/j.diabet.2010.10.009

[8] Nakanishi N, Okamoto M, Yoshida H, Matsuo Y, Suzuki K, et al (2003) Serum uric acid and risk for development of hypertension and impaired fasting glucose or Type II diabetes in Japanese male office workers. Eur J Epidemiol 18: 523–530. 12908717 10.1023/a:1024600905574

[9] Meisinger C, Döring A, Stöckl D, Thorand B, Kowall B, et al (2012) Uric acid is more strongly associated with impaired glucose regulation in women than in men from the general population: the KORA F4-Study. PLoS One 7: e37180. 22615932 10.1371/journal.pone.0037180 PMC3353894

[10] Kawamoto R, Tabara Y, Kohara K, Kusunoki T, Abe M, et al (2013) Serum uric acid is more strongly associated with impaired fasting glucose in women than in men from a community-dwelling population. PLoS One 8: e65886. 23785457 10.1371/journal.pone.0065886 PMC3681777

[11] Kodama S, Saito K, Yachi Y, Asumi M, Sugawara A, et al (2009) Association between serum uric acid and development of type 2 diabetes. Diabetes Care 32: 1737–1742. 19549729 10.2337/dc09-0288 PMC2732137

[12] Anton FM, Garcia Puig J, Ramos T, Gonzalez P, Ordas J (1986) Sex differences in uric acid metabolism in adults: evidence for a lack of influence of estradiol–17 beta (E2) on the renal handling of urate. Metabolism 35: 343–348. 3959904 10.1016/0026-0495(86)90152-6

[13] Sumino H, Ichikawa S, Kanda T, Nakamura T, Sakamaki T (1999) Reduction of serum uric acid by hormone replacement therapy in postmenopausal women with hyperuricaemia. Lancet 354: 650. 10466673 10.1016/S0140-6736(99)92381-4

[14] World Health Organization: Definition and diagnosis of diabetes mellitus and intermediate hyperglycemia. Geneva: Warld Health Organization, 2006.

[15] Kuzuya T, Nakagawa S, Satoh J, Kanazawa Y, Iwamoto Y, et al (2002) Report of the committee on the classification and diagnostic criteria of diabetes mellitus. Diabetes Res Clin Pract 55: 65–85. 11755481 10.1016/s0168-8227(01)00365-5

[16] Yamada T, Fukatsu M, Suzuki S, Wada T, Yoshida T, et al (2010) Fatty liver predicts impaired fasting glucose and type 2 diabetes mellitus in Japanese undergoing a health checkup. J Gastroenterol Hepatol 25: 352–356. 19817963 10.1111/j.1440-1746.2009.05998.x

[17] Unwin N, Shaw J, Zimmet P, Alberti KG (2002) Impaired glucose tolerance and impaired fasting glycaemia: the current status on definition and intervention. Diabet Med 19: 708–723. 12207806 10.1046/j.1464-5491.2002.00835.x

[18] Chen KT, Chen CJ, Gregg EW, Imperatore G, Narayan KM (2003) Impaired fasting glucose and risk of diabetes in Taiwan: follow-up over 3 years. Diabetes Res Clin Pract 60: 177–182. 12757990 10.1016/s0168-8227(03)00037-8

[19] Moebus S, Stang A, Möhlenkamp S, Dragano N, Schmermund A, et al (2009) Association of impaired fasting glucose and coronary artery calcification as a marker of subclinical atherosclerosis in a population-based cohort-results of Heinz Nixdorf Recall Study. Diabetologia 52: 81–89. 18979083 10.1007/s00125-008-1173-y

[20] Kato M, Noda M, Suga H, Matsumoto M, Kanazawa Y (2009) Fasting plasma glucose and incidence of diabetes–implication for the threshold for impaired fasting glucose: results from the population-based Omiya MA cohort study. J Atheroscler Thromb 16: 857–861. 20032586 10.5551/jat.1792

[21] Moriuchi T, Oka R, Yagi K, Miyamoto S, Nomura H, et al (2010) Diabetes progression from “high-normal” glucose in school teachers. Intern Med 49: 1271–1276. 20606358 10.2169/internalmedicine.49.3513

[22] Miyake T, Kumagi T, Hirooka M, Koizumi M, Furukawa S, et al (2012) Metabolic markers and ALT cutoff level for diagnosing nonalcoholic fatty liver disease: a community-based cross-sectional study. J Gastroenterol 47: 696–703. 22331365 10.1007/s00535-012-0534-y

[23] Miyake T, Kumagi T, Hirooka M, Furukawa S, Koizumi M, et al (2013) Body mass index is the most useful predictive factor for the onset of nonalcoholic fatty liver disease: a community-based retrospective longitudinal cohort study. J Gastroenterol 48: 413–422. 22933183 10.1007/s00535-012-0650-8

[24] Kojima S, Watanabe N, Numata M, Ogawa T, Matsuzaki S (2003) Increase in the prevalence of fatty liver in Japan over the past 12 years: analysis of clinical background. J Gastroenterol 38: 954–961. 14614602 10.1007/s00535-003-1178-8

[25] American Diabetes Association. Screening for Diabetes (2003) Diabetes care. 24 Suppl. 1 s21–24.

[26] Harita N, Hayashi T, Sato KK, Nakamura Y, Yoneda T, et al (2009) Lower serum creatinine is a new risk factor of type 2 diabetes: the Kansai healthcare study. Diabetes Care 32: 424–6. 19074997 10.2337/dc08-1265 PMC2646021

[27] Jimba S, Nakagami T, Takahashi M, Wakamatsu T, Hirota Y, et al (2005) Prevalence of nonalcoholic fatty liver disease and its association with impaired glucose metabolism in Japanese adults. Diabet Med 22: 1141–5. 16108839 10.1111/j.1464-5491.2005.01582.x

[28] Sairenchi T, Iso H, Nishimura A, Hosoda T, Irie F, et al (2004) Cigarette smoking and risk of type 2 diabetes mellitus among middle-aged and elderly Japanese men and women. Am J Epidemiol 160: 158–62. 15234937 10.1093/aje/kwh183

[29] Waki K, Noda M, Sasaki S, Matsumura Y, Takahashi Y, et al (2005) JPHC Study Group. Alcohol consumption and other risk factors for self-reported diabetes among middle-aged Japanese: a population-based prospective study in the JPHC study cohort I. Diabet Med 22: 323–31. 15717882 10.1111/j.1464-5491.2004.01403.x

[30] Khosla UM, Zharikov S, Finch JL, Nakagawa T, Roncal C, et al (2005) Hyperuricemia induces endothelial dysfunction. Kidney Int 67: 1739–1742. 15840020 10.1111/j.1523-1755.2005.00273.x

[31] Roy D, Perreault M, Marette A (1998) Insulin stimulation of glucose uptake in skeletal muscles and adipose tissues in vivo is NO dependent. Am J Physiol 274: E692–E699. 9575831 10.1152/ajpendo.1998.274.4.E692

[32] Sautin YY, Nakagawa T, Zharikov S, Johnson RJ (2007) Adverse effects of the classic antioxidant uric acid in adipocytes: NADPH oxidase-mediated oxidative/nitrosative stress. Am J Cell Physiol 293: C584–C596. 10.1152/ajpcell.00600.2006 17428837

[33] Schulz E, Gori T, Münzel T (2011) Oxidative stress and endothelial dysfunction in hypertension. Hypertens Res 34: 665–73. 21512515 10.1038/hr.2011.39

[34] Smelcerovic A, Rangelov M, Smelcerovic Z, Veljkovic A, Cherneva E, et al (2013) Two 6-(propan-2-yl)-4-methyl-morpholine-2,5-diones as new non-purine xanthine oxidase inhibitors and anti-inflammatory agents. Food Chem Toxicol 55: 493–7. 23410588 10.1016/j.fct.2013.01.052

[35] Quinones-Galvan A, Natali A, Baldi S, Frascerra S, Sanna G, et al (1995) Effect of insulin on uric acid excretion in humans. Am J Physiol 268: E1–E5. 7840165 10.1152/ajpendo.1995.268.1.E1

[36] Johnson RJ, Perez-Pozo SE, Sautin YY, Manitius J, Sanchez-Lozada LG, et al (2009) Hypothesis: could excessive fructose intake and uric acid cause type 2 diabetes? Endocr Rev 30: 96–116. 19151107 10.1210/er.2008-0033 PMC2647706

[37] Choi HK, Liu S, Curhan G (2005) Intake of purine-rich foods, protein, and dairy products and relationship to serum levels of uric acid: the Third National Health and Nutrition Examination Survey. Arthritis Rheum 52: 283–9. 15641075 10.1002/art.20761

